# Population Screening of Gambling Behavior: Playing to Escape From Problems May Be a Key Characteristic of At-Risk Players

**DOI:** 10.3389/fpsyt.2021.690210

**Published:** 2021-09-01

**Authors:** Klavdia Neophytou, Marios Theodorou, Christiana Theodorou, Tonia-Flery Artemi, Georgia Panayiotou

**Affiliations:** Department of Psychology and Center for Applied Neuroscience, University of Cyprus, Nicosia, Cyprus

**Keywords:** gambling, risk, population screening, gender, motivation, escape, avoidance, emotion regulation

## Abstract

The increase in gambling availability and the inclusion of gambling disorder as an addiction in DSM-5 highlight the importance of brief screening measures aiming to identify at-risk gamblers. The current study, using a brief telephone survey, assessed demographic characteristics and gambling behaviors in 2,118 adults. Questions were developed based on DSM-5 criteria for Gambling Disorder and common assessment tools. A 7% prevalence of as at-risk gamblers was identified. Male gender, low monthly income, high frequency of gambling behavior, large amounts of money spent, and gambling to escape from everyday problems or for amusement, specifically for men, were found to be the characteristics that can help in the early identification of at-risk gamblers. Gambling for financial gain and as a way to socialize, age, and employment status were not significant predictors of gambling severity. This study shows that the above characteristics can be assessed easily through phone screening of large populations, aiding in prevention practices to reduce the problematic use of gambling activities.

## Introduction

Increased availability of gambling opportunities and the expansion of legalized gambling have been identified as an important public health concern (1) by many countries. At the same time, the inclusion of Gambling Disorder as a Behavioral Addiction in DSM-5 (2) instigated the need to understand the psychopathology of this condition and risk factors for its development, while many national authorities have focused their policy-making on enhancing healthy, regulated gambling vs. pathological engagement with this behavior.

The increase in gambling is true also for the Republic of Cyprus, a European country with a population of 875,899. Increased prevalence of gambling in Cyprus is indicated by the fact that there are >506 betting stores, i.e., at least 1 per 1,731 citizens, when considering only this form of gambling activity (personal communication, National Betting Authority).

Only one unpublished epidemiological study, conducted with face-to-face interviews on a random sample of the population, examined gambling behavior in Cyprus (3), which was funded by the country's National Betting Authority, in an attempt to characterize and describe gambling in this country. The study was descriptive, providing initial frequency estimations of problematic gambling, and showing that 13% of Cypriots present probable problematic gambling behavior, with 6% identified as problematic gamblers based on a self-report questionnaire using DSM-5 criteria. The two most cited reasons why Cypriots engage in gambling activities in this study were for financial gain (60%) and amusement (49%). The three top choice gambling activities were the Cyprus government scratch lottery (79%), other lotteries (68%), and bingo (47%).

At the international level, in an effort to understand and respond better to this public health challenge, various individual characteristics including demographic, e.g., gender, age, and socioeconomic status [e.g., (4)]; comorbid psychopathology, e.g., alcohol abuse and depression [e.g., (5)]; cognitive distortions, e.g., illusion of control [e.g., (6)]; personality characteristics, e.g., impulsivity [e.g., (7)]; frequency of gambling activities [e.g., (8)]; and gambling motives [e.g., (9)] have been examined as factors that might best predict the development and/or maintenance of problematic gambling behavior [see review by Dowling et al. (10)].

None of these dimensions have been previously examined in Cyprus, while even in the international literature, much research remains to be done on the unique and interactive contribution of these factors in explaining gambling disorder risk. To inform policy makers both in Cyprus and elsewhere, and implement public policy strategies that safeguard the population from developing this addiction, it is important to be able to identify characteristics of at-risk players through fast, cost-effective population screening that can be repeated at regular time intervals in order to implement measures when risky behaviors increase. Accurate screening can lead to improved care and reduced healthcare cost (11).

A meta-analysis by Dowling et al. (12) underscores the importance of short screening population measures for gambling based on DSM criteria. Extending these suggestions, the present study utilizes a large, telephone survey that incorporates demographics and individual traits, in addition to gambling disorder symptoms, so as to identify not only those who already experience symptoms but also those at risk for developing them. Therefore, the study aims to enhance the understanding of gambling behavior and contribute to the international literature by providing data that help in the identification of key characteristics that describe at-risk players, and can be assessed using brief screening procedures.

The screening tool was constructed for purposes of this study, drawing from existing knowledge of risk factors for gambling addiction and DSM-5 diagnostic criteria. Based on the literature, demographic characteristics including male gender (10), young age (4), low income (13), and employment status (14) are considered as risk factors for gambling severity. Additionally, it seems that higher gambling severity is characterized by high frequency of gambling as well as higher amounts of money spent (8). However, these characteristics increase risk when combined with particular motives for gambling. While typical, non-pathological gamblers seem to engage in this behavior for purposes of socialization, excitement, and amusement, problem gamblers seem to engage in gambling mainly for other reasons, including avoidance of problems and financial gain (15). We hoped to verify that motives, in combination with specific demographic characteristics, indeed differentiate between gamblers who experience gambling-related problems, as per their self-report, and those who do not, in this under-researched setting.

To summarize, the aim of the current study was to describe the characteristics of individuals who self-report that their gambling behavior has been causing problems in their daily life in order to contribute to the identification of important demographic predictors of those at-risk to develop serious dysfunction and gambling addiction. A secondary aim was to provide preliminary descriptive data on the gambling behavior of the adult population in Cyprus through brief telephone screening.

## Materials and Methods

### Participants

The sample was composed of all adults living in all regions under the jurisdiction of the Republic of Cyprus, over 18 years old, who had a telephone and could be reached via random number dialing. The telephone survey was conducted via the NIPO/CATI software between November 2019 and early March 2020 and took about 10 min. For inclusion, participants had to answer “yes” to the first question, which asked if they had played a gambling game at least once in their lifetime (a list of games was provided, including the option “other” for the cases when none of the listed games were endorsed). People with zero involvement (i.e., who denied ever playing any gambling game) were excluded from participation but were asked if another adult in their household was present at the time who was over 18 and participated in gambling sometimes. A total of 25,919 phone numbers were dialed, which were either selected randomly from phone directories (of mainly stationary numbers) or were randomly generated numbers of mobile phones. From this pool, 15,223 people answered the phone call. From those who answered, a total of 2,118 participated in the phone survey. The majority of those who answered the phone were excluded: most (12,794) refused to participate when they were informed that they were called about a study, 58 refused when they were informed about the General Data Protection Regulation (i.e., how anonymity would be kept), 14 were younger than 18 years old, and 239 reported zero gambling behavior. The final sample was *N* = 2,118 (1,242 male; Mean_age_ = 48 years, SD = 15, Mode = 36). Of the total, 70% were employed, 15% retired, 9% unemployed, 4% students, 1.3% recipients of welfare, and 0.4% were soldiers. About 10% of the sample reported no income, 6% reported a gross monthly income <500 euro, 56% reported 500–3,500 euro, 4% reported >3,500 euro, and 8% did not answer.

### Measure

Questions were developed based on DSM-5 criteria for Gambling Disorder, and lists of gambling activities were derived from common assessment tools, i.e., the South Oaks Gambling Screen—Revised (16) and the Gambling Commission website, adjusted for the cultural context. Questions pertained to demographics, gambling behavior, problems due to gambling, and gambling motives (see Table 1; Cronbach's alpha for frequency and money spent = 0.91, for 15 gambling activities = 0.74, for five questions referring to problem gambling = 0.97, and gambling motives = 0.68). In sum, the risk factors we examined were as follows: (a) gender, (b) age based on date of birth, (c) income, (d) employment status, (e) frequency of gambling behavior, (f) amount of money spent on gambling, and (g) reasons for gambling. All were examined with a small number of questions as part of a short population screening tool.

**Table 1:** Screening questions and answers.

**1**	***Gender:* Male/Female**
2	*Year of birth:* ………………………….
3	*Employment status:* Unemployed/Employed in the public spectrum/Employed in the private spectrum/Self-employed/Soldier/Student/Recipient of welfare support
4	*Monthly income (euro):* <500/501–1,000/1,001–1,500/1,501–2,000/2,001–2,500/2,501–3,000/3,001–3,500/More than 3,500/No income/Do not know or want to answer
5	“*Which of these gambling games have you ever played in your life?” (A list of gambling activities was mentioned)* Casino games/Card games/Dice games/Government scratch lottery/Other lotteries/Sports betting/Horse races/Bingo/Kazanti (traditional game)/Online sports betting/Online casino/Slot machines/Skill games for money (e.g., basketball)/Participation in a draw/Stock market/Other
6	“*How often you were involved with gambling activities the last year?”* Once or more than once a week/Once or more than once a month/Once or more than once every 3 months/Once or more than once every 6 months/Once or more than once a year/Do not know or want to answer
7	“*How much money do you usually spend on gambling activities every month the last year?” (euro)* 1–30/31–100/101–1,000/1,001–10,000/more than 10,000/Do not know or want to answer
8	“*Have you ever felt the need to bet more money on gambling the last year?”* YES/NO/Do not know or want to answer
9	“*Have you even bet more money that you could afford the last year?”* Never/Sometimes/Most of the times/Almost always/Do not know or want to answer
10	“*The last year, have even been any cases where a family member or a friend has criticized you for your gambling behavior or told you that you are likely to engage in it to a degree that is problematic, regardless of whether you agreed or disagreed with that?”* Never/Sometimes/Most of the times/Almost always/Do not know or want to answer
11	“*Have you felt that your level of gambling has caused you problems or has a negative effect on you in the last year?”* Never/Sometimes/Most of the times/Almost always/Do not know or want to answer
12	“*Have you ever had to lie to people who are important to you in the last year about the amount of money you bet or the frequency which you gamble?”* YES/NO/Do not know or want to answer
13	“*What do you think that your involvement with gambling offers to you?”* a)Entertainment b)Economic profit c)Way of socialization d)A way to escape from the problems of everyday lifee) Other

### Data Analysis

First, frequency analysis was done to characterize the sample's gambling behavior and motives. Second, to identify the subgroup of participants who are at risk for problem gambling, a total score was derived from five items assessing DSM-5-based symptoms, with one question each: (a) need for increased gambling, (b) betting more money than one can afford, (c) receiving criticism about one's gambling, (d) experiencing problems or negative consequences due to gambling, and (e) having to lie about one's gambling. Items were assessed on a 0–3 scale (never experienced to almost always) or 0–1 (no or yes). Total scores on this *problem gambling inde*x ranged from 0 to 6, *M* = 1.12, *SD* = 0.49. To address questions about the characteristics of individuals at risk, participants who scored 2 and above and therefore more DSM symptoms (1 *SD* from the Mean; *N* = 148 participants, representing 7% of the total sample) were considered as at-risk gamblers. Next, Pearson correlations were performed to examine the associations among the main variables assessed by the questionnaire including problem gambling index, monthly income, frequency, money spent, and motives. After that, a comparison analysis was done between at-risk gamblers and the rest of the sample (low-risk gamblers; *N* = 1,970) on demographic variables, gambling behavior, and gambling motives. Chi-square analysis was used when the dependent variable was dichotomous and Mann–Whitney *U*-test when the dependent variable was ordinal.

## Results

### Description of Gambling Behavior in Cyprus

About 23% of the participants reported gambling at least once a week, 24% at least once a month, 16% at least once every 3 months, 13% at least once every 6 months, and 21% at least once a year (3% no answer). Of the participants, 73% spent 1–30 euro every month for gambling the last year, 15% spent 30–100 euro, 6.8% spent 100–1,000 euro, and 1% spent 1,000–10,000 euro (3.7% no answer). To the question which games/betting activities they ever engaged in (from a list of 15 activities, plus “other” category), the most frequently reported activities were as follows: state scratch lottery by 86% of participants, other lotteries 83%, and bingo 50%. These were followed by kazanti (traditional numbers game played at fares) 46%, sports betting 25%, stock market 20.6%, participation in a draw 20.6%, casino games 20%, card games (e.g., poker) 19.5%, slot machines 17.5%, online sports betting 12.3%, and horse races 8.6%, The three least frequently reported activities were dice games 5%, skill games for money (e.g., basketball) 4.6%, and online games, including online casino, 4%. No additional games were reported to the “other” option, with the few reported answers corresponding to subcategories of the above 15 options.

### Gambling Motives

To the question regarding reasons for gambling, participants could freely give multiple responses. The most frequent reasons were given in order as follows: amusement 39%, financial gain 31%, socialization 3%, and a way to escape everyday problems 2%. Thirty-one percent also noted “other” reasons, which, however, largely overlapped with the above categories (e.g., “adrenaline,” “happiness,” and “to pass time”), and therefore, their responses were allocated in the previous categories.

### Correlation Between Variables

Table 2 presents the Pearson's r correlation among the study variables. In accordance with our prediction, gambling to escape from problems was the only gambling motive that was significantly association with problematic gambling behavior.

**Table 2:** Correlation between gambling levels of involvement, demographics, gambling behaviors, and motives.

**Variable**	**1**	**2**	**3**	**4**	**5**	**6**	**7**	**8**
1. Monthly income								
2. Frequency	−0.01							
3. Money spent	−0.02	0.06^*^						
4. Problem gambling index	−0.5^*^	0.17^**^	0.08^**^					
5. Amusement	−0.01	−0.04	0.01	0.04				
6. Financial	−0.03	0.20^**^	0.02	0.01	−0.35^**^			
7. Way of socialization	−0.02	−0.01	−0.03	0.01	−0.02	−0.04		
8. Escape everyday problems	−0.01	0.01	−0.01	0.10^**^	−0.04	−0.01	0.06^*^	

** *Correlation is significant at the 0.01 level*.

* *Correlation is significant at the 0.05 level*.

### Characteristics of Gamblers Reporting Problem Behavior

Chi-square test of differences between at-risk and low-risk gamblers showed a significant effect of (a) *gender*χ^2^ (1, *N* = 2,017) = 24.13, *p* < 0.0001; (b) reporting *amusement* as a reason for gambling χ^2^ (1, *N* = 2,017) = 4.49, *p* < 0.05; and (c) reporting *escape from everyday problems* as a reason for gambling χ^2^ (1, *N* = 2,017) = 20.52, *p* < 0.0001 (see Table 3 for all the expected and count cells).

**Table 3:** Level of gambling involvement based on gender and gambling motives.

		**Gender**			
		**Female**		**Male**	
		**Count**	**Expected**	**Count**	**Expected**
Level of gambling	Risk	60	96	172	136
Involvement	Low risk	767	731	996	1,032
		**Amusement**			
		**Mentioned**		**Not mentioned**	
		**Count**	**Expected**	**Count**	**Expected**
Level of gambling	Risk	98	91	134	141
Involvement	Low risk	686	693	1,077	1,070
		**Way to escape from everyday problems**			
		**Mentioned**		**Not mentioned**	
		**Count**	**Expected**	**Count**	**Expected**
Level of gambling	Risk	16	5	216	227
Involvement	Low risk	25	36	1,738	1,727

However, group differences in reporting *financial gain* (*p* = 0.23) and *socialization* (*p* = 0.50) as reasons for gambling were not statistically significant.

For those who mentioned amusement and escape as reasons for gambling, chi-square test of difference between at-risk and low-risk gambles showed a significant effect of gender separately for every reason: (a) amusement χ^2^ (1, *N* = 784) = 8.38, *p* < 0.005; and (b) escape from everyday problems, χ^2^ (1, *N* =41) = 5.66, *p* < 0.05. In both cases, these motives were reported more frequently by men (see Table 4 for all the expected and count cells).

**Table 4:** Level of gambling involvement based on the combination of gender and gambling motives.

**Mentioned Amusement as a reason for gambling**					
		**Female**		**Male**	
		**Count**	**Expected**	**Count**	**Expected**
Level of gambling	Risk	26	39	72	58
Involvement	Low risk	287	273	399	412
**Mentioned gambling as a way to escape from everyday problems**					
		**Female**		**Male**	
		**Count**	**Expected**	**Count**	**Expected**
Level of gambling	Risk	1	4	15	12
Involvement	Low risk	10	7	15	18

A Mann–Whitney test showed that at-risk gamblers had significantly lower monthly income (Mdn = 777) than the low-risk group (Mdn = 942), *U* = 97,023, *p* < 0.0001, η^2^ = 0.01; were involved in gambling significantly more often (Mdn = 1380 vs. Mdn = 970), *U* = 79770, *p* < 0.0001, η^2^ = 0.03; and spent significantly more money (Mdn = 1,398) than the low-risk group (Mdn = 966), *U* = 77,105, *p* < 0.0001, η^2^ = 0.07. However, there was no significant group difference on year of birth, *p* = 0.52, or employment status, *p* = 0.62.

## Discussion

This study helps to identify characteristics that distinguish at-risk gamblers, while also providing descriptive information on gambling behavior in Cyprus, using a brief and easy population screening approach. Descriptive findings regarding demographic factors involved in gambling severity were largely similar to what was found in a previous epidemiological study (3), suggesting that valid descriptions of the gambling situation can be obtained through a brief, low-cost telephone survey. Importantly, this study compares at-risk gamblers with low-risk gamblers, based on established DSM-5 criteria, aiming to identify key parameters that might help identify problem gambling during large population screening, as a way of early problem identification.

The number of participants who identified as at-risk gamblers (7%) is in agreement with the previous epidemiological study (3), which identified 6% of the participants as pathological/problematic gamblers; it also agrees with that study in terms of the most common gambling activities of Cypriots. In Europe, problem gambling rates seem to be between 0.7 and 6.5% (17), which provides validation to the current estimate. It should be noted that the obtained information, as intended, represents screening, showing some level of problematic consequences of gambling in a portion of the population, that can be used in prevention efforts. Percentages of individuals meeting the clinical criteria for gambling disorder may be substantially lower and would require thorough individualized clinical assessment to be recognized. As noted in Calado and Griffiths' (17) review of worldwide prevalence, rates of pathological gambling are lower than the percentage of at-risk gamblers, around 1%, consistent with DSM-5 estimates.

From the present data, it appears, as would be expected from the previous literature, that male gender, low income, frequent gambling, and spending more money on gambling correlate with and best separate at-risk and low-risk gamblers. Male gender and high gambling frequency have been previously related with problem gambling (8, 18), while high income has been related to fewer gambling problems (19). Additionally, it came as no surprise that spending higher amounts of money on gambling activities is an important risk factor, as one of the DSM criteria is the “need to gamble with increasing amounts of money in order to achieve the desired excitement” (2).

In contrast, year of birth (age range) and employment status did not differentiate significantly between at-risk and low-risk gamblers in this sample. Generally, age presents contradictory results in the literature, as it has been found as an important risk factor in some studies (18) but not in others (19). Employment status has also mostly yielded non-significant results in the literature, consistent with present findings (14, 18, 19);. Taken together, these data provide credibility to the fact that risk factors identified through a quick screening of the general population can reliably identify problematic gambling, similar to much more extensive surveys.

The main contribution of this study is to add to the existing evidence that specific types of gambling motives strongly differentiate between at-risk and low-risk gamblers. Gambling as a way of amusement characterized primarily low-risk gamblers, while gambling as a way to escape from everyday problems characterized mainly at-risk gamblers. As noted in the study by Ricketts and Macaskill (15), “normal” gamblers were much more likely than problem gamblers to report finding alternative ways to improve negative emotions, and they also consistently reported gambling for entertainment, in accord with present results. Findings also agree with several studies showing that gambling is sustained by both positive and negative reinforcement through increasing pleasant and decreasing unpleasant emotions (20–22), but these two pathways seem to characterize at-risk and low-risk gamblers to different degrees. The results of the current study suggest that using gambling as an escape from everyday problems may be a strong correlate of problem gambling, even more so that using this activity for amusement, a finding that agrees with a recent review, and a substantial body of recent studies, showing that problem gambling behavior is often maintained as an emotion regulation strategy in the absence of more adaptive ways to cope (23).

Being able to identify this risk factor even within the context of a brief screening adds to its validity as a potentially critical characteristic of at-risk populations, corresponding to criterion 5 of the DSM-5 description of gambling disorder. This finding highlights the role of dysfunctional emotion regulation and coping as an important factor to consider in public health measures, as it is documented by much emerging literature and contemporary models of gambling addiction, as a core component of the psychopathology of this disorder (24–26).

Additionally, the results show that gambling for amusement and escape from problems characterizes mostly at-risk men rather than at-risk women, in accordance with Francis et al. (27). This interactive effect deserves further research, however, as contradictory evidence exists, e.g., McGrath et al. (28) who found that women are more likely to gamble as a way to regulate mood and escape problems and 26 who showed that coping motives for gambling were related mostly to women and enhancement motives to men. Flack and Stevens (29) instead found no difference between men and women with regards to gambling motives. Our finding, however, is consistent with the well-accepted evidence that men turn more frequently than women to substances to cope with difficulties (30, 31)—more research is needed to validate if this holds reliably in the case of gambling. However, the imbalanced sample of participants (men, women) in favor of men in our current sample may have affected the results, suggesting some caution in interpreting this interaction.

Gambling as a way of socialization and financial gain did not significantly distinguish at-risk gamblers. Gambling for socialization has been found to be less strongly related with problem gambling previously (32). Gambling to win money was one of the popular motives of gambling involvement in the current study and has been listed as an important reason of problematic gambling in the literature (33). Making money from gambling was viewed as desirable by both normal and problem gamblers in the study by Ricketts and Macaskill (15), but not as a credible outcome by the normal gamblers, suggesting that it is not the desire to earn financial gain that may distinguish problem gamblers, but the distorted perception of the likelihood of this happening, something that was not assessed in the current study. Findings agree with Flack and Morris (2015), who showed that the most predominant gambling motives of problem gamblers are emotion-focused instead of directed at financial gain; although financial motives are one of the most frequent self-reported motives, it is not the one that best identifies problematic patterns of gambling.

This study comes with some limitations that include the self-reported nature of questions and using a screening tool, developed for this study, which, however, relied on questions formatted after well-established measures and the DSM-5. In particular, the telephone survey method does not allow for any objective measures against which to validate whether the respondents indeed face gambling problems. In defense of the current design, this is likely not different from other forms of self-report measures, while the similarity in findings obtained in relation to the previous door-to-door interview study conducted in the same population speaks to the validity of our method. However, future studies need to consider a combination of established self-report questionnaires and clinician measures to further validate the utility of such short screening tools. One more limitation of the study is the absence of information regarding those who refused to participate in the study in order to compare them with those who participated (all participants refused either when they were informed about the purpose of the phone call or when they were informed about General Data Protection regulation, therefore not being able to collect any data at any of these points).

In spite of these shortcomings, this study shows that male gender, low monthly income, high frequency of gambling behavior, large amounts of money spent, and gambling as escape and amusement specifically for men are characteristics that can help in the early identification of at-risk gamblers, and that these can be assessed easily through phone screening of large populations, so that prevention practices can be implemented to reduce the problematic use of gambling activities.

## Data Availability Statement

The raw data supporting the conclusions of this article will be made available by the authors, without undue reservation.

## Ethics Statement

The studies involving human participants were reviewed and approved by Cyprus National Bioethics Committee. Written informed consent for participation was not required for this study in accordance with the national legislation and the institutional requirements.

## Author Contributions

KN: supervision of data collection, analyzing data, and writing. MT, CT, and T-FA: supervision of data collection. GP: supervision of the whole project and writing. All authors contributed to the article and approved the submitted version.

## Conflict of Interest

The authors declare that the research was conducted in the absence of any commercial or financial relationships that could be construed as a potential conflict of interest.

## Publisher's Note

All claims expressed in this article are solely those of the authors and do not necessarily represent those of their affiliated organizations, or those of the publisher, the editors and the reviewers. Any product that may be evaluated in this article, or claim that may be made by its manufacturer, is not guaranteed or endorsed by the publisher.
